# Effects of Cationic Polyacrylamide Characteristics on Sewage Sludge Dewatering and Moisture Evaporation

**DOI:** 10.1371/journal.pone.0098159

**Published:** 2014-05-30

**Authors:** Jun Zhou, Fenwu Liu, Chengyi Pan

**Affiliations:** 1 College of Biotechnology and Pharmaceutical Engineering, Nanjing Tech University, Nanjing, China; 2 Environmental Engineering Laboratory, College of Resource and Environment, Shanxi Agricultural University, Taigu, China; University of Kansas, United States of America

## Abstract

The effects of the molecular weight (MW) and charge density (CD) of cationic polyacrylamide (CPAM) on sludge dewatering and moisture evaporation were investigated in this study. Results indicated that in sludge conditioning, the optimum dosages were 10, 6, 6, 4, and 4 mg g^−1^ CPAM with 5 million MW and 20% CD, 5 million MW and 40% CD, 3 million MW and 40% CD, 8 million MW and 40% CD, and 5 million MW and 60% CD, respectively. The optimum dosage of CPAM was negatively correlated with its CD or MW if the CD or MW of CPAM was above 20% or 5 million. In the centrifugal dewatering of sludge, the moisture content in the conditioned sludge gradually decreased with the extension of centrifugation time, and the economical centrifugal force was 400×g. The moisture evaporation rates of the conditioned sludge were closely related to sludge dewaterability, which was in turn significantly correlated either positively with the solid content of sludge particles that were >2 mm in size or negatively with that of particles measuring 1 mm to 2 mm in diameter. During treatment, sludge moisture content was reduced from 80% to 20% by evaporation, and the moisture evaporation rates were 1.35, 1.49, 1.62, and 2.24 times faster in the sludge conditioned using 4 mg g^−1^ CPAM with 5 million MW and 60% CD than in the sludge conditioned using 4 mg g^−1^ CPAM with 8 million MW and 40% CD, 6 mg g^−1^ CPAM with 5 million MW and 40% CD, 6 mg g^−1^ CPAM with 3 million MW and 40% CD, and 10 mg g^−1^ CPAM with 5 million MW and 20% CD, respectively. Hence, the CPAM with 5 million MW and 60% CD was ideal for sludge dewatering.

## Introduction

Currently, the amount of wastewater treated by wastewater treatment plants in China exceeds 125,000,000 m^3^ per day. As a result, approximately 6,000,000 dry tons of sewage sludge is generated each year [1]. This amount increases substantially with the development of wastewater treatment facilities. Thus, proper avenues of sewage sludge disposal must be developed urgently [2]–[3]. The maximum moisture content in sewage sludge often reaches ∼99% after secondary sedimentation and 95% to 98% after thickening in wastewater treatment plants [4]–[5]. This high moisture content is a major issue in sludge disposal because it increases transport and storage costs, decreases energy efficiency during incineration, requires increased amounts of supplemental bulking agents during composting, and increases leachate concentrations in sludge landfill [6]–[7]. Therefore, the reduction of the moisture content in sludge by either dewatering or evaporation is a critical issue in sludge disposal.

Prior to dewatering, sewage sludge is typically flocculated with polyelectrolytes to enhance dewaterability. Polyelectrolytes are conventionally used in sludge conditioning, which mainly involves inter-particle bridging and surface charge neutralization [8]–[9]. In inter-particle bridging, the polyelectrolyte chains are adsorbed on the solid surface, and form loops, trains, and tail configurations. When two sludge particles merge, the loops and tails of one particle adhere to bare patches on the approaching particle to form bridges. The effectiveness of bridge flocculation is directly related to molecular weight (MW) [10]. In surface charge neutralization mechanism, flocculation occurs because the electrostatic force of repulsion between the charged sludge particles is reduced. Therefore, polyelectrolytes with high charge density (CD) form flocs effectively. The zeta potential of the sludge system can be used to determine the change in surface charge during polyelectrolyte flocculation. Kleimann et al. [11] found that optimum flocculation occurs when the polyelectrolyte dose is sufficient to lower the zeta potential to almost zero.

Cationic polyacrylamide (CPAM) is a type of polyelectrolyte that has been widely used as a sludge conditioner because of its low dosage requirement, high efficiency, and pollution-free effects. Previous studies report that CPAM dosage is critical in sludge conditioning because overdoses increase cost and deteriorate sludge dewaterability [12]. Selected specific sludge reach the optimal dewatering point when they are conditioned with 15 mg g^−1^ of CPAM (dry basis), according to Luo et al. [13]. However, the dewatering point deteriorates drastically when CPAM dosage reaches 20 mg g^−1^. As indicated above, the MWs and CDs of polyelectrolytes are important in the flocculation of sewage sludge before dewatering. However, their influence on optimum CPAM dosage in sludge dewatering has not been examined previously. Sludge drying is a necessary intermediate process common to all sludge disposal methods because it reduces the water content by a large margin, resulting in a large reduction in sludge mass and volume. Sludge can be dried either by natural air drying at a working temperature of 20°C to 30°C [14]–[15] or by thermal drying at temperatures above 100°C [16]–[17]. Regardless of the drying technology used, the moisture evaporation behavior of sludge is the key factor that controls the drying effect; faster moisture evaporation rates indicate better sludge drying [18]–[19]. Nonetheless, information regarding the evaporation behavior of sludge moisture is limited, particularly after maximum sludge dewatering with different types of CPAM at optimum dosages. Crucial data on the influence of MWs and CDs on optimum CPAM dosage or sludge moisture evaporation behavior at optimal dewatering points with different types of CPAM at optimum dosages is also lacking, which strongly limits the accurate evaluation of additional CPAM in optimal sludge dewatering.

In view of this information, this study aims to: (1) determine the optimum CPAM dosage for sludge conditioning at different MWs and CDs; and (2) explore the sludge moisture evaporation behavior at optimal dewatering points with different types of CPAM at optimum dosages.

## Materials and Methods

### Ethics Statement

No specific permits were required for the described field studies and no specific permissions were required for these locations. The location is not privately-owned or protected in any way.

### Sampling of Sewage Sludge

The sewage sludge used in this study was obtained from the Loufan Wastewater Treatment Plant in Taiyuan City of Shanxi Province in China. The plant receives mostly domestic sewage from the city. For the sludge dewatering trials, mixed sludge was obtained from the primary and secondary settling tanks of a sludge thickening pond. The samples were collected in polypropylene bottles and maintained at 4°C before use. Immediately after collection, the pH level and specific resistance to filtration (SRF) of the sludge samples were determined. Moisture content was measured by oven drying at 105°C. In the dried sludge samples, organic matter content was measured according to the methods prescribed by the American Public Health Association [20]. The physiochemical properties of the selected municipal sewage sludge were as follows: pH 6.82±0.02; moisture content 98.00±0.04%; organic matter content 57.3±0.1% (dry basis); and SRF 2.05±0.02×10^13 ^m kg^−1^.

### Preparation of CPAM Solutions

In this study, five types of CPAM were used as sludge conditioners. These CPAM types were characterized as follows: CPAM with 5 million MW and CDs of 20%, 40%, and 60%; 3 million MW and CD of 40%; and 8 million MW and CD of 40% (Gongyi City Qiyuan Chemical Industry Co., Ltd.). For each type of CPAM, stock solutions (0.2%) were prepared with distilled water and used after 24 h.

### Design of Experiments

#### Sludge conditioning

200 mL samples of original sewage sludge were conditioned in 500 mL beakers with either 2, 4, 6, 8, 12, 16, 20, 24, or 28 mL (1, 2, 3, 4, 6, 8, 10, 12, or 14 mg g^−1^, dry basis) of CPAM solutions with (1) 5 million MW and 20% CD in experiment series 1; (2) 5 million MW and 40% CD in experiment series 2; (3) 5 million MW and 60% CD in experiment series 3; (4) 3 million MW and 40% CD in experiment series 4; and (5) 8 million MW and 40% CD in experiment series 5. All treatments were administered in triplicate. The sludge in each beaker was stirred using a magnetic stirrer (85-2B, Jintan Medical Instrument Factory, China) at 300 r min^−1^ for the first 20 s and then at 100 r min^−1^ for an additional 40 s. After sludge conditioning, water was added because the CPAM solutions were removed from the systems immediately. The SRFs of the conditioned sludge were then measured in all treatments.

The SRF data indicated that the optimum doses were 4, 4, 6, 6, and 10 mg g^−1^ (dry basis) for CPAM with 5 million MW and 60% CD, 8 million MW and 40% CD, 5 million MW and 40% CD, 3 million MW and 40% CD, and 5 million MW and 20% CD, respectively. Through the following treatment, particle size distribution and the zeta potential of conditioned sludge were determined: (1) Sludge dewatering capability improved gradually after the original sludge was conditioned using CPAM with 5 million MW and 60% CD at concentrations of 1, 2, 3, and 4 mg g^−1^ (dry basis) as a representative process; and (2) the original sludge was conditioned with different types of CPAM at the optimum dosages.

#### Centrifugal sludge dewatering efficiency after CPAM conditioning

In this study, two series experiments were conducted. In the first experiment, the original sludge was conditioned using CPAM with 5 million MW and 60% CD at concentrations of 1, 2, 3, and 4 mg g^−1^. The conditioned sludge was then dewatered in a centrifuge (TDL-40B, Shanghai Precision Instrument Co., Ltd.) at 1600×g for 300 s. In the second experiment, the original sludge was conditioned with different types of CPAM at optimum doses as previously described. The conditioned sludge was dewatered using the centrifugal method at 100, 400, 900, or 1600×g for 180 s. The moisture content of the centrifugally dewatered sludge and the solid content in the resulting supernatant were then examined. All treatments were performed in triplicate.

#### Moisture evaporation characteristics of sludge after CPAM conditioning

During the drying processes of unconditioned sludge and of sludge that was optimally conditioned using different types of CPAM at maximum dosages, the moisture evaporation rates were obtained by placing 50 mL of each of the different sludge samples on petri dishes (87 mm in diameter, 14 mm high). These dishes were then moved to a thermostatic oven (GZX-9246 MBE, Shanghai Boxun Industry and Commerce Co., Ltd, China) at 25°C with an air flow rate of 1.5 m s^−1^ for 2 d (2880 min). The moisture evaporation rates were determined based on the moisture content in sludge during different time periods. All treatments were performed in triplicate.

#### Analytical Methods

SRF was determined using the Buchner funnel method [21]–[22]. The size distribution of sludge particles was determined using the sieving method described by Laguna et al [23]. Zeta potential of sludge was recorded on a micro-electrophoresis machine (Model JS94H, Shanghai, China) [1]. The moisture content of the centrifugally dewatered sludge and the solid content of the resultant supernatant were measured by oven-drying at 105°C [24]. Moisture content in the sludge during the moisture evaporation process was calculated with the moisture content in the original sludge and the weight of the sludge over time.

#### Statistical Analyses

Data were analyzed with SPSS (SPSS 20.0 for Windows). There were three samples were determined to calculated the means and standard deviations. All of the figures presented include the standard deviations of the data and were drawn with Origin 7.5 software.

## Results and Discussion

### SRF of Sludge after Conditioning with Different Types of CPAM

Sludge SRF is widely used to assess sludge dewaterability [25]–[26]. Generally, a high SRF value indicates poor sludge dewaterability [25].

**Figure 1:**
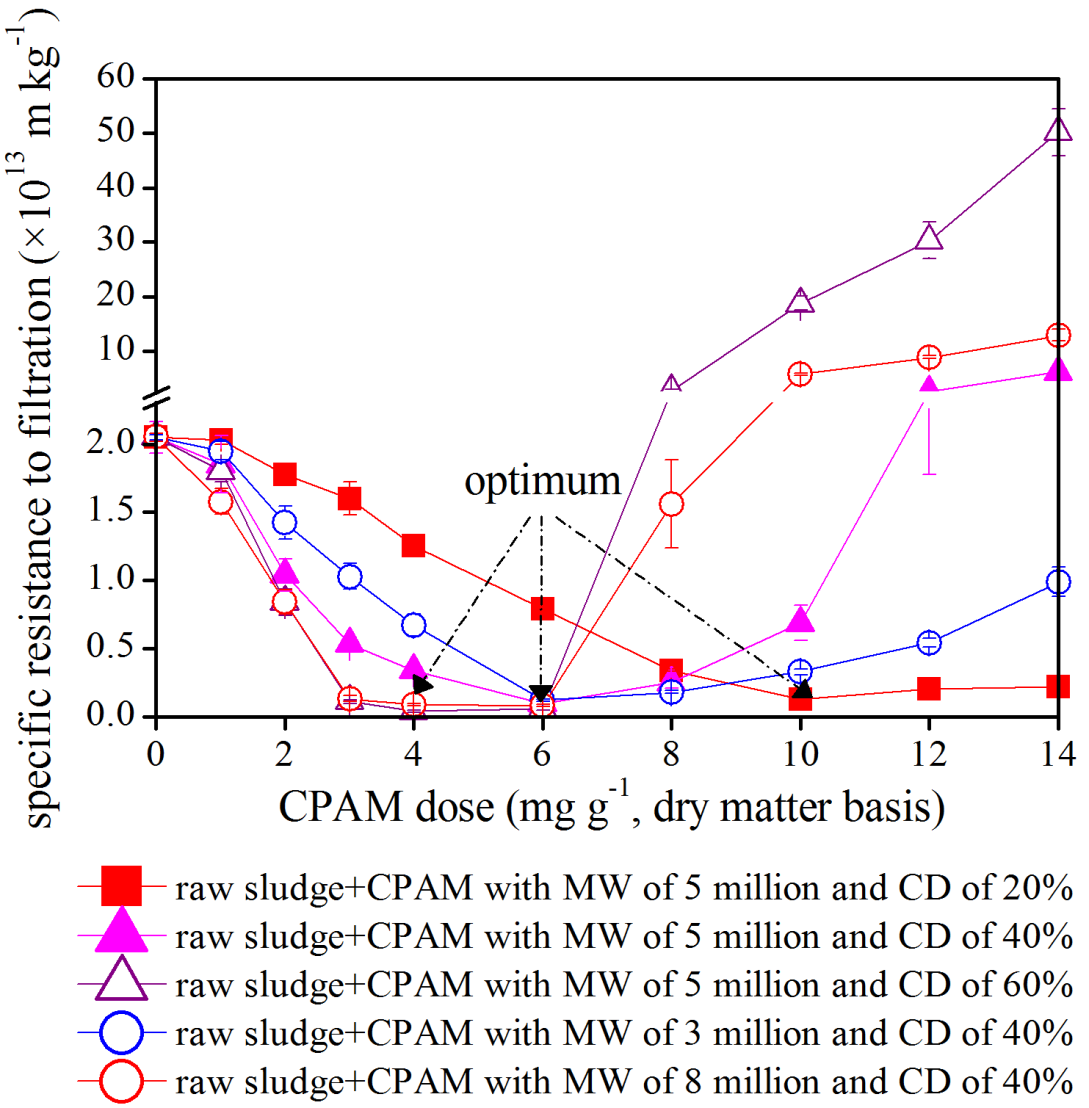
Effect of different CPAM dosages on specific resistance to filtration (SRF) of sewage sludge.

The variations in sludge SRF after conditioning with different types of CPAM are presented in Fig. 1. The SRF of the original sludge is 2.05×10^13 ^m kg^−1^, and this value was initially reduced by the additions of all types of CPAM. However, the SRF gradually increased again when the optimum CPAM dosages were obtained. This result implies that any additional increase beyond the optimum polyelectrolyte dose deteriorates sludge dewaterability in all treatment groups. This observation is in agreement with the results obtained by Chang et al [12]. For example, the optimal dose of CPAM with 5 million MW and 60% CD was 4 mg g^−1^, resulting in SRF of 0.04×10^13 ^m kg^−1^. However, SRF increased to 0.06×10^13^, 2.67×10^13^, 18.8×10^13^, 30.3×10^13^, and 50.2×10^13 ^m kg^−1^ when 6, 8, 10, 12, and 14 mg g^−1^ of CPAM were added, respectively. The current study determines valuable that had not been reported in previous studies, such as the optimum dose of CPAM for sludge conditioning given different MWs and CDs. In sludge dewatering, the optimum doses were 10, 6, 6, 4, or 4 mg g^−1^ for CPAM with 5 million MW and 20% CD, 5 million MW and 40% CD, 3 million MW and 40% CD, 8 million MW and 40% CD, or 5 million MW and 60% CD, respectively. At this optimum dewatering point, sludge SRF was either 0.13×10^13^, 0.10×10^13^, 0.12×10^13^, 0.08×10^13^, or 0.04×10^13 ^m/kg. These values could increase to 0.22×10^13^, 6.20×10^13^, 0.90×10^13^, 12.9×10^13^, or 50.2×10^13 ^m kg^−1^ when the amount of CPAM reached 14 mg g^−1^ in the above corresponding systems.

In sludge dewatering, the optimum dosage of CPAM was negatively correlated with its CD (*R^2^ = 0.96*). For instance, the optimum dosage of CPAM with 5 million MW decreased from 10 mg g^−1^ to either 6 mg g^−1^ or 4 mg g^−1^ when CD increased from 20% to either 40% or 60%, respectively. The optimum dosage of CPAM and its MW are negatively correlated if the MW of CPAM was above 5 million. Hence, the optimum dosage of CPAM with 40% CD declined from 6 mg g^−1^ to 4 mg g^−1^ when MW increased from 5 million to 8 million. However, the optimum dosage of CPAM was remained at 6 mg g^−1^ when the MW shifted between 3 and 5 million. In other words, the optimum dosage of CPAM was negatively correlated with its CD or MW if the CD or MW of CPAM was above 20% or 5 million.

### Particle Size Distribution and Zeta Potential of Sludge after Conditioning with Different Types of CPAM

**Figure 2:**
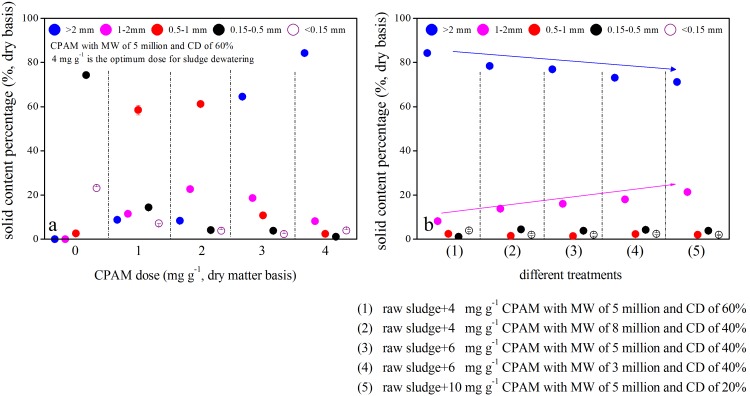
Effect of CPAM doses and types on sludge particle size distribution.

The effect of CPAM dosage and type on sludge particle size distribution during sludge conditioning is plotted in Fig. 2. The size distribution of sludge particles is affected by the dosage of CPAM in conditioning (Fig. 2a). In the present study, the percentages of solid content in raw sludge were 2.6%, 74.3%, and 23.1% (dry basis) in 0.5 mm to1 mm, 0.15 mm to 0.5 mm, and <0.15 mm particle size ranges, respectively. The particle size of raw sludge was below 1 mm, and most particles measured between 0.15 mm and 0.5 mm. This finding is consistent with the conclusions obtained by Wilén and Balmér [27] and Defrance et al [28]. Both reports indicated that the diameter of activated sludge particles is smaller than 1 mm and that these particles are mostly distributed in the range of 0.1 mm to 0.6 mm. The particle size of sludge gradually increased with the increase in CPAM dosage to the optimum dosage range. For example, the percentages of solid content in the conditioned sludge with particles larger than 2 mm were 8.3%, 8.8%, 64.6%, and 84.3% when 1, 2, 3, and 4 mg g^−1^ of CPAM with 5 million MW and 60% CD were added, respectively (Fig. 2a).

When sludge was optimally conditioned through CPAM with different MWs and CDs, particle size distributions varied significantly (Fig. 2b). The solid content of sludge with particle sizes measuring >2 mm and 1 mm to 2 mm were 84.3% and 8.2%, 78.5% and 13.7%, 76.9% and 16.0%, 73.0% and 18.0%, or 71.1% and 21.2% in sludge conditioned using 4 mg g^−1^ CPAM with 5 million MW and 60% CD, 4 mg g^−1^ CPAM with 8 million MW and 40% CD, 6 mg g^−1^ CPAM with 5 million MW and 40% CD, 6 mg g^−1^ CPAM with 3 million MW and 40% CD, or 10 mg g^−1^ CPAM with 5 million MW and 20% CD, respectively. In this study, the SRF of the sludge in corresponding systems was either 0.04×10^13^, 0.08×10^13^, 0.10×10^13^, 0.12×10^13^, or 0.13×10^13 ^m kg^−1^. As unambiguously presented above, there is a negative significant correlation between solid content of sludge with particles >2 mm in diameter and SRF of conditioned sludge (*R^2^ = 0.99*). However, the solid content of sludge with particles that are 1 mm to 2 mm in diameter is positively correlated with the SRF of conditioned sludge (*R^2^ = *0.98). It is observed that solid content of sludge with particles smaller than 1 mm almost have no differences (from 7.1% to 9.0%) in the different treatments.

**Figure 3:**
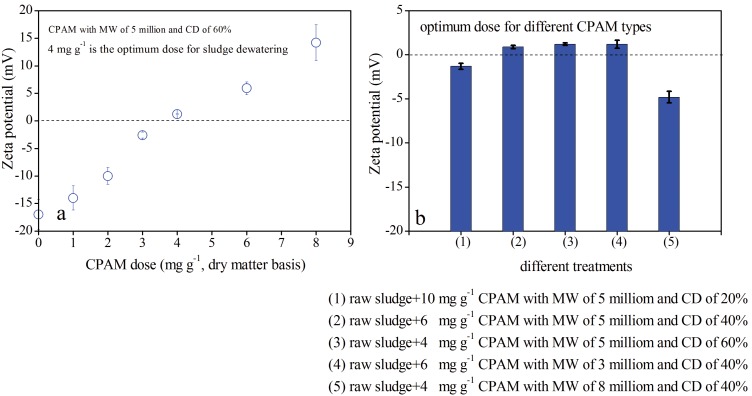
Effect of CPAM doses and types on sludge zeta potential.

Fig. 3 shows the zeta potential of sludge after conditioning with different CPAM types at various doses. It is indicated that zeta potential gradually increased with CPAM concentration. As shown in Fig. 3a, the zeta potential of raw sludge in the system increased from −17.0 mV to 14.2 mV when the amount of CPAM with 5 million MW and 60% CD was increased from 0 mg g^−1^ to 8 mg g^−1^. The raw sludge underwent optimal conditioning when the amount of CPAM with 5 million MW and 60% CD reached 4 mg g^−1^ and when the zeta potential of sludge was 1.2 mV. These results are in agreement with those of Kleimann et al. [11], who observed that optimum flocculation is achieved when the polyelectrolyte dose is sufficient to lower the zeta potential to almost zero. The addition of CPAM with MW of 5 million and CD of 40%, MW of 3 million and CD of 40%, MW of 8 million and CD of 40%, and MW of 5 million and CD of 20% at their optimum doses, changed the zeta potential to 0.9, 1.2, −4.8 and −1.3 mV, respectively (see Fig. 3b).

### Centrifugal Dewatering Efficiency of Sludge after Conditioning with Different Types of CPAM

**Figure 4:**
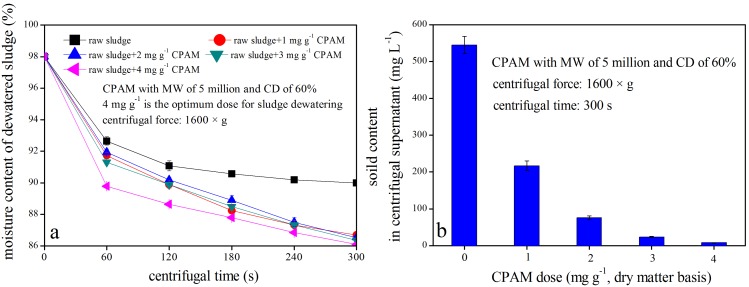
Centrifugal dewatering efficiency of sludge after conditioning with CPAM.

To evaluate the efficiency of different sludge dewatering technologies, the moisture content in dewatered sludge and the solid content in centrifugal supernatants are typically considered [29]–[30]. Fig. 4 displays the efficiency of centrifugal sludge after conditioning using CPAM with 5 million MW and 60% CD.

During centrifugal dewatering, the moisture content in dewatered sludge, particularly conditioned sludge, gradually decreased with the extension of centrifuging time (Fig. 4a). For instance, the moisture content in dewatered sludge decreased from 98.0% to 92.6%, 91.0%, 90.6%, 90.2%, or 90% in unconditioned sludge after centrifuging at 1600×g for 60, 120, 180, 240, or 300 s, respectively. However, the moisture content of sludge conditioned using 4 mg g^−1^ CPAM with 5 million MW and 60% CD was reduced to 89.8%, 88.6%, 87.8%, 86.8%, or 86.1% at the corresponding time point. As shown in Fig. 4a, the better the sludge conditioning, the lower the moisture content in dewatered sludge. However, the solid content in the supernatants was 217, 76, 24, or 8 mg L^−1^ in the corresponding treatments (Fig. 4b). Based on the SRF data shown in Fig. 1, sludge SRF in the corresponding systems was either 1.80×10^13^, 0.84×10^13^, 0.11×10^13^, or 0.04×10^13 ^m kg^−1^. Thus, the solid contents in the supernatants was positively correlated with sludge SRF (*R^2^ = 0.98*).

**Figure 5:**
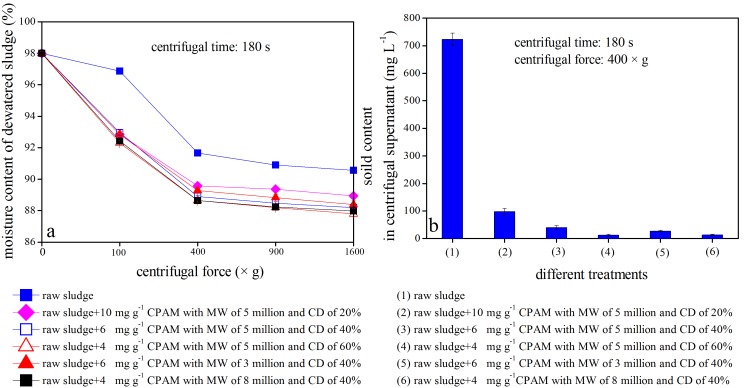
Centrifugal dewatering efficiency of sludge after conditioning with different CPAM types at optimum doses.

Fig. 5 shows the sludge dewatering efficiency after conditioning with different types of CPAM at optimum doses. The moisture contents in both raw and conditioned sludge declined drastically when the centrifugal speed increased from 0×g to 400×g for 180 s (Fig. 5a). The moisture content in dewatered sludge was maintained even when the centrifugal speed increased further from 400×g to 1600×g. This finding indicates that 400×g is the economical centrifugal speed for the centrifugal dewatering of conditioned sludge. As indicated in Fig. 5b, the solid contents in the supernatants was either 12, 13, 40, 27, or 98 mg L^−1^ when the sludge was conditioned using 4 mg g^−1^ CPAM with 5 million MW and 60% CD, 4 mg g^−1^ CPAM with 8 million MW and 40% CD, 6 mg g^−1^ CPAM with 5 million MW and 40% CD, 6 mg g^−1^ CPAM with 3 million MW and 40% CD, or 10 mg g^−1^ CPAM with 5 million MW and 20% CD, respectively, when all of these samples were centrifuged at 400×g for 180 s. Based on the SRF value provided previously, sludge SRF in the corresponding systems was either 0.04×10^13^, 0.08×10^13^, 0.10×10^13^, 0.12×10^13^, or 0.13×10^13 ^m kg^−1^. In general, the proportion of solid particles in supernatants declined with the improvement of sludge dewaterability after centrifugal sludge dewatering.

### Moisture Evaporation Behavior of Sludge after Conditioning with Different Types of CPAM

**Figure 6:**
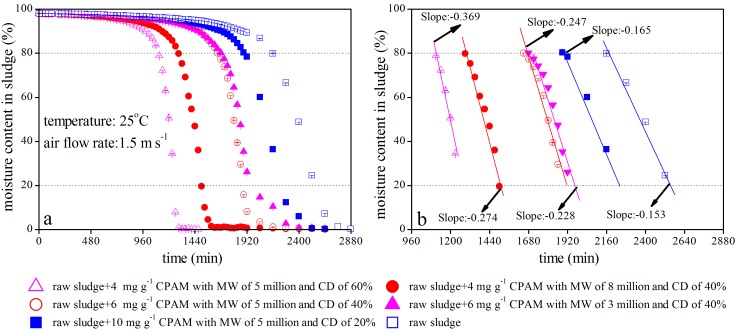
Moisture evaporation behavior of sludge conditioned with different CPAM types at optimum doses.

Fig. 6 illustrates the moisture evaporation behavior of sludge conditioned with different CPAM types at optimum doses. The curves in Fig. 6a show that all of the sludge samples were almost completely dried within 2880 min at 25°C given an air flow rate 1.5 m s^−1^. However, total drying time differed with the various CPAM types and optimum doses applied to the sludge. The total drying times for sludge samples conditioned with different types of CPAM at optimum doses were shorter than those for untreated sludge. The water in sludge is typically divided into two categories: free and bound water.

The evaporation of bound water consumes more heat than that of free water [30]–[31], indicating that a gradual shift from bound water to free water occurs with the addition of CPAM. This theory was confirmed by Katsiris [32], who reported that cationic polyelectrolytes cause a reduction in sludge bound water content. This reduction is attributed to the replacement of water molecules in the sludge by the adsorbed coagulant.

The moisture evaporation rate of conditioned sludge could follow a sequence of fast to slow, as demonstrated in sludge conditioned using 4 mg g^−1^ CPAM with 5 million MW and 60% CD, 4 mg g^−1^ CPAM with 8 million MW and 40% CD, 6 mg g^−1^ CPAM with 5 million MW and 40% CD, 6 mg g^−1^ CPAM with 3 million MW and 40% CD, and 10 mg g^−1^ CPAM with 5 million MW and 20% CD (Fig. 6a). This finding implies that free water content may differ in sludge conditioned with different types of CPAM at optimum doses.

Moisture evaporation rates may also have a close relationship with sludge particle size >2 mm or particles 1∼2 mm in diameter. The higher the proportion of particle size >2 mm and the lower the proportion of particle size 1∼2 mm in diameter, the faster the evaporation rate of conditioned sludge. This observation is exemplified by the particle size distributions and moisture evaporation rates in sludge conditioned with various CPAMs at the optimum dosage. This result mirrors that of Feng et al. [25], who concluded that the evaporation rates sludge moisture may be slowed when the sludge contains numerous tiny particles because large particle sizes in a substance increase the total surface area for water adhesion [33]. Such adhesion hinders the free transfer of water from the bottom of the sludge mass to the top during the drying process.

The moisture content in conventional dewatered sludge is often as high as 80% [1], [7], [34]. Therefore, moisture evaporation rates below 80% are of general concern in sludge management. A significant linear correlation was observed between sludge moisture content and drying time when the sludge moisture content decreased from 80% to 20% during the evaporation process (as shown in Fig. 6b). The moisture evaporation rates in sludge conditioned using 4 mg g^−1^ CPAM with 5 million MW and 60% CD were 1.35, 1.49, 1.62, 2.24, and 2.40 times faster in reducing the moisture content from 80% to 20% than in sludge conditioned using 4 mg g^−1^ CPAM with 8 million MW and 40% CD, 6 mg g^−1^ CPAM with 5 million MW and 40% CD, 6 mg g^−1^ CPAM with 3 million MW and 40% CD, 10 mg g^−1^ CPAM with 5 million MW and 20% CD, and the original sludge. These rates can be determined from the slope of the fitting line provided in Fig. 6b. Although the optimum dosage for sludge dewatering was 4 mg g^−1^, CPAM with 5 million MW and 60% CD was more effective than the CPAM with 8 million MW and 40% CD in term of sludge conditioning and dewatering because the moisture evaporation rate of the former was faster than that of the latter. Similarly, the CPAM with 5 million MW and 40% CD was also superior to the CPAM with 3 million MW and 40% CD. The CPAM with MW of below 8 million or 60% CD was typically used in sludge dewatering given the relatively cheap cost of this CPAM, according to survey data from the Jiangsu and Shanxi wastewater treatment plants. In other words, CPAM cost increases significantly if the CPAM either has an MW above 8 million or 60% CD. In turn, the cost of sludge dewatering increases drastically as well. For instance, the price of CPAM with 8 million MW and 60% CD is 16, 000 RMB per ton. However, the price will increases to 24, 000 RMB per ton if the MW and CD of CPAM are 12 million and 80% (Please see Table S1). Therefore, the CPAM type with either an MW above 8 million or a 60% CD is not discussed in this study.

## Conclusion

In sludge dewatering, the results of this study reveal that the optimum doses were 10, 6, 6, 4, and 4 mg g^−1^ for CPAM with 5 million MW and 20% CD, 5 million MW and 40% CD, 3 million MW and 40% CD, 8 million MW and 40% CD, and 5 million MW and 60% CD, respectively. Sludge SRF also decreased to 0.13×10^13^, 0.10×10^13^, 0.12×10^13^, 0.08×10^13^, and 0.04×10^13 ^m kg^−1^, respectively. Generally, a high SRF value indicates poor sludge dewaterability, the moisture content in conditioned sludge gradually decreased with the extension of centrifugation time during centrifugal dewatering, and the economical centrifugal speed was 400×g. In centrifugal sludge dewatering, the proportion of solid particles in supernatants decreases with the improvement of sludge dewaterability. In addition, sludge dewaterability was either positively correlated with the solid content of conditioned sludge with particles >2 mm in diameter or negatively correlated with the solid content of sludge particles measuring 1 mm to 2 mm in diameter. The proportion of solid particles exists in supernatants is in turn closely associated with the moisture evaporation rates of the conditioned sludge. When sludge moisture content was reduced from 80% to 20% by evaporation in each treatment, the moisture evaporation rates in the sludge conditioned using 4 mg g^−1^ CPAM with 5 million MW and 60% CD were 1.35, 1.49, 1.62, and 2.24 times faster than those in sludge conditioned using 4 mg g^−1^ CPAM with 8 million MW and 40% CD, 6 mg g^−1^ CPAM with 5 million MW and 40% CD, 6 mg g^−1^ CPAM with 3 million MW and 40% CD, and 10 mg g^−1^ CPAM with 5 million MW and 20% CD, respectively. Thus, the CPAM with 5 million MW and 60% CD is ideal for sludge dewatering.

## Supporting Information

Table S1 **The price of CPAM with different molecular weight (MW) and charge density (CD) (doc).** This material is available free of charge via the Internet at http://www.plosone.org.
(DOC)
