# Supplementary material for: Effects of Cationic Polyacrylamide Characteristics on Sewage Sludge Dewatering and Moisture Evaporation
Source: PLoS One. 2014 May 30;9(5):e98159. doi: 10.1371/journal.pone.0098159 (PMC4039440; doi:10.1371/journal.pone.0098159)
Supplement: Table S1 — The price of CPAM with different molecular weight (MW) and charge density (CD) (doc). This material is available free of charge via the Internet at http://www.plosone.org. (DOC) [file pone.0098159.s001.doc]

**Supplemental information**

According to survey data from the Jiangsu and Shanxi wastewater treatment plants in China, the price of CPAM with different molecular weight (MW) and charge density (CD) has been given in here (Table S1) as supplemental information.

Table S1 the price of CPAM with different molecular weight (MW) and charge density (CD)

| Molecular weight (MW) | Charge density (CD) | Price (RMB per ton) |
| --- | --- | --- |
| 3~8 million | 20~60% | 13, 000~16, 000 |
| 12 million | 60% | 20, 000 |
| 8 million | 80% | 22, 000 |
| 12 million | 80% | 24, 000 |
